# A New and Effective Procedure for Advanced Oral Cancer Therapy: The Potential of a Cancer Stem Cell Assay in Guiding Chemotherapy

**DOI:** 10.37825/2239-9747.1042

**Published:** 2023-12-08

**Authors:** Francesca Spirito, Pier Paolo Claudio, Candace M. Howard, Jagan Valluri, Krista L. Denning, Lorenzo Lo Muzio, Antonio Cortese

**Affiliations:** a Department of Clinical and Experimental Medicine, University of Foggia, Italy; b Department of Pharmacology and Toxicology, Cancer Center & Research Institute, University of Mississippi Medical Center, Jackson, MS 39216, USA; c Department of Radiology, University of Mississippi Medical Center, Jackson, MS 39216, USA; d Department of Biological Sciences, Translational Genomics Research Institute, Marshall University, Huntington, WV 25701, USA; e Department of Pathology, Joan C. Edwards School of Medicine, Marshall University, Huntington, WV 25701, USA; f Department of Medicine, Surgery, and Dentistry, Unit of Maxillofacial Surgery, University of Salerno, 84084, Italy

**Keywords:** ChemoID, Head & neck cancer, Oral cancer, Oral squamous cell carcinoma, Chemotherapy, Individualized therapy, Personalized medicine

## Abstract

**Introduction:**

Ineffective anticancer therapy can result in unnecessary toxicity and the development of resistant clones. Many types of solid tumors, including head and neck squamous cell carcinoma, have been found to contain a small population of cancer stem cells (CSCs) that contribute to tumor propagation, maintenance, and treatment resistance.

**Materials and methods:**

Selectively enriched CSCs from primary cancer cell cultures can be used in a chemosensitivity assay for a functional test (ChemoID) that uses patients’ live tumor cells to indicate which chemotherapy agent (or “combinations”) will kill not only the bulk of tumor cells but also the CSCs that are known to cause cancer to recur. This study aimed to show the potential of testing the sensitivity of CSCs enriched from oral cancer patients’ biopsies to conventional chemotherapies. A case series of eleven patients affected by advanced oral squamous cell carcinoma (OSCC) have been included in this study. We compared the results of the CSC assay among all the patients and found that there was variability in the chemotherapy response predicted by the assay.

**Results:**

Variability in chemotherapy response was found by the CSC assay in advanced OSCC patients suggesting more precise and personalized therapies to the Oncologist.

**Conclusions:**

Variability in chemosensitivity for OSCC warrants the need to investigate further the use of the assay in larger cohorts to gain a broader understanding of the utility of the clinical test.

## 1. Introduction

Oral cancer is the 11th most common malignancy in the world [1]. Although it is widely spread throughout the world, 70 % of oral cancers are diagnosed in developing countries [1]. The most common type of this cancer is squamous cell carcinoma, which represents 5 % of male and 1 % of female tumors. The geographical distribution of this malignant tumor is strongly influenced by risk factors, but the overall incidence of oral carcinoma is 16.1 for every 100,000 adults [1,2].

Tumors of the oral cavity may also be secondary to primary cancer from other body sites. Mandibular metastases occur in cases of breast cancer, lung cancer, clear cell renal cell carcinoma, and prostate cancer [3].

Treatment protocols of OSCC include combinations of surgical resection of the primary tumor and eventual lymph nodal dissection of the neck that may be followed by radiation therapy. Chemotherapy is added as a complementary treatment to radiation therapy when extracapsular metastatic lymph node extension is suspected.

Common chemotherapy protocols for advanced head & neck cancer include cisplatin monotherapy or the combination of cisplatin with 5-Fluorouracil or anti-EGF, such as cetuximab. Unfortunately, patients with the same stage and grade of cancer may vary considerably in their clinical response to chemotherapy and ineffective anticancer therapy can result in unnecessary toxicity and the development of resistant clones.

Cancer stem cells (CSCs) have been identified in OSCC as well as in several other solid malignant tumors [4–6]. CSCs possess the ability of self-renewal and proliferation, producing progenitor cells and cancer cells that drive tumor growth [7]. CSCs are known to be responsible for metastasis, drug resistance, and cancer recurrence. Thus, controlling CSCs may provide an effective therapeutic intervention that inhibits tumor growth and aggressiveness. For this reason, it is essential to diagnose this disease early and to target treatment for each patient, in order to improve their survival prognosis.

ChemoID is a functional assay capable of identifying the most effective chemotherapy cocktail able to kill the cancer stem cells (CSCs), which are responsible for cancer recurrence and metastatic spread [8–11].

This leads to individualized chemotherapy based on the patient’s specific tumor characteristics.

The aims of this study were to: 1) develop and optimize protocols for the disinfection and international shipment of fresh biopsy samples, and 2) to investigate the clinical potential of the ChemoID assay in predicting the sensitivity of OSCC to first-, second-, and third-line chemotherapies. Eleven patients affected by advanced oral squamous cell carcinoma (OSCC) have been included in this study. We compared the results of the ChemoID assay among this patients’ cohort and found that they had a variability in the chemotherapy response predicted by the assay, which justifies the use of the diagnostic assay to identify the most effective treatment.

## 2. Materials and methods

### 2.1. Chemotherapy agents

The agents investigated for this OSCC cohort with the ChemoID® assay as single chemotherapy or in combination were: carboplatin, cisplatin, paclitaxel, 5-fluorouracil, bleomycin, methotrexate, cetuximab, docetaxel. However, the assay is versatile, and other chemotherapy agents were tested such as vinorelbine, ifosfamide, gemcitabine, and erlotinib.

### 2.2. OSSC biopsy, and shipping modalities

To minimize contamination of the fresh biopsies, we have developed a system to decontaminate the biopsies and send the samples to the clinical laboratory in the United States of America that performed the ChemoID assay.

For each patient, two tissue biopsy samples were collected in the operating room under sterile conditions. The first incisional biopsy in patients affected by oral cancer was sent to the local Pathology Departmental laboratory of the San Giovanni di Dio Ruggi d’Aragona University Hospital in Salerno (Italy), in 5 % formaldehyde solution to confirm the histological diagnosis.

The second biopsy specimen to be shipped to the ChemoID laboratory was disinfected a second time using a Betadine solution followed by irrigation with a sterile saline solution to remove the Betadine disinfectant and it was placed in a sterile vial containing a transportation media (provided by the clinical laboratory).

In the case of highly contaminated biopsy sites of the oral cavity, which more often are in extensively ulcerated OSCC, with difficult or impossible stable disinfection during the biopsy procedure, we adopted an oblique technique to prevent contamination of the biopsy.

After the first incisional biopsy, another specimen was collected by using a trocar at the same site of the incisional biopsy after disinfection with Betadine at the site of the incision. Through the deep insertion of the trocar needle, we prevented the sample from contamination by microorganisms in the oral cavity.

The samples for the ChemoID assay were shipped same-day by FedEx clinical pack express delivery using a polystyrene box, carefully packed and temperature-controlled to maintain room temperature during transportation. The packaging was in full compliance with the international standards for the safety and durability of biological shipments. A 2.2 mm thick lead sheet was wrapped around the shipping vial to protect the specimens from X-ray exposure by airport scanners, ensuring tissue viability at arrival.

### 2.3. ChemoID assay

The ChemoID assay is a Clinical Laboratory Improvement Amendments (CLIA)-certified and College of American Pathologists (CAP)-accredited test that is performed in the clinical pathology laboratories of the Cabell Huntington Hospital and Edwards Cancer Center in West Virginia, USA.

ChemoID is an assay that evaluates the response of various chemotherapies as single agents or in combination on cancer stem cells and the bulk of tumor cells by measuring cell survival after 48 h from chemotherapy treatments [8–11].

To generate the primary tumor cell cultures, which contain the bulk of tumor cells, the fresh tumor tissue from surgical biopsies was minced and gently disassociated in a biosafety cabinet. The CSCs were enriched from the primary tumor cell cultures using a 3D-suspension cell culture rotating bioreactor with a gas-permeable membrane that allows for gas exchange. Culture media, oxygenation, rotation speed, temperature, and CO2 were kept consistently constant in an incubator. The bioreactor can rotate at adjustable speed on a fixed axis creating a 3D-suspension cell culture in the absence of shear forces. Primary cells were counted and 2 × 10^6 cells were cultured in the bioreactor for 7- days set at 25 rpm with airflow set at 20 % in RPMI media in the absence of growth factors. Plates (96-well) were seeded with equal numbers of either bulk tumor cells or CSCs and incubated at 37 °C. After 24 h, clinical-grade chemotherapy drugs were added alone or in combination for 1-h exposure. After the 1-h exposure, the treatment media containing the various chemotherapies were removed and replaced with fresh media. Cell viability was assessed 48 h later as previously. For each treatment, percent survival (potential therapeutic efficacy) was calculated relative to appropriate controls. Efficacy and resistance of each drug and combinations were reported on the ChemoID assay results as a continuous number from <10 % to 100 % cell-kill.

Time lapse from sample shipping to getting ChemoID results ranged 15–21 days, not impacting on results. Moreover, chemotherapy could immediately start following standard protocol and be redirected into personalized scheme after ChemoID result gaining under Oncologist decision.

The cancer cells and cancer stem cells are challenged with doses of chemotherapy equal to the Cmax found in the serum of patients treated with clinical doses of the chemotherapy treatment.

### 2.4. Cohorts of patients

After signing an informed consent, eleven subjects affected by oral squamous cell carcinoma (OSCC) were included in the study (Table 1). Demographic, clinical, and radiographic information were collected from the database of the Department of Maxillo-Facial and Oral Surgery of the San Giovanni di Dio Ruggi d’Aragona Hospital University in Salerno, Italy.

## 3. Results

### 3.1. Results of the ChemoID assay

The results of the assay from the eleven patients affected by squamous cell carcinoma of the oral cavity are reported in Table 2. The percentage of cell kill response of the CSCs to the panel of chemotherapeutic agents tested by the ChemoID assay was unique for each patient, although the tumor histological type was the same.

The results from the assay demonstrated variability of chemotherapy response predicted among patients affected by the same histological type of oral cancer. Sensitivity to chemotherapy did not appear to depend on the site of the squamous cell carcinoma. In fact, Case #1 and Case #9 with squamous cell carcinoma of the tongue responded differently to chemotherapy. In particular, Case #9 was highly responsive (78.6 %) to cisplatin 100 mg/m^2^, whereas Case #1 was only moderately responsive (34.2 %).

Other instances of individual sensitivity not linked to the site of the tumor was found for cases 2 and 4 of oral mucosa. Case #2 was highly responsive (87.4 %) to docetaxel 75 mg/m^2^, whereas Case #4 was only moderately responsive (29.2 %), however, both cases were sensitive to cisplatin 100 mg/m^2^ (73.6 % and 85.3 %, respectively).

We compared the response of the chemotherapies tested at clinical doses on samples with the same histological tumor diagnosis of OSCC. Table 3 reports the chemotherapies and the dose to which the CSCs of the various patients were most sensitive.

The results of the assay shown in Table 3, indicate that there was often a greater response when chemotherapy agents were used in combination (particularly cisplatin, 5-fluorouracil, and docetaxel) than when they were tested as monotherapy.

### 3.2. Case presentation

**Fig. 1:**
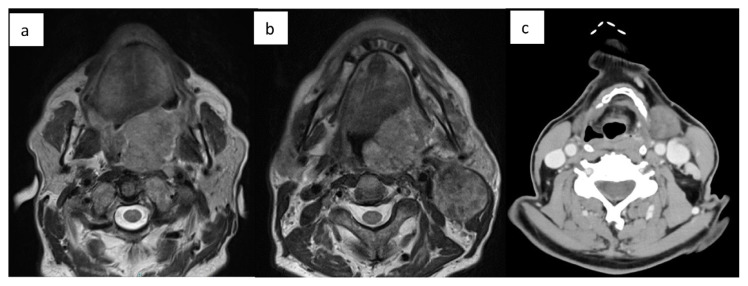
MRI and CT axial images before chemotherapy. a) MRI image with an axial cut on the lingual plane; b) MRI image with an axial cut on the mandibular plane; c) CT image with an axial cut on the hyoid bone plane.

**Fig. 2:**
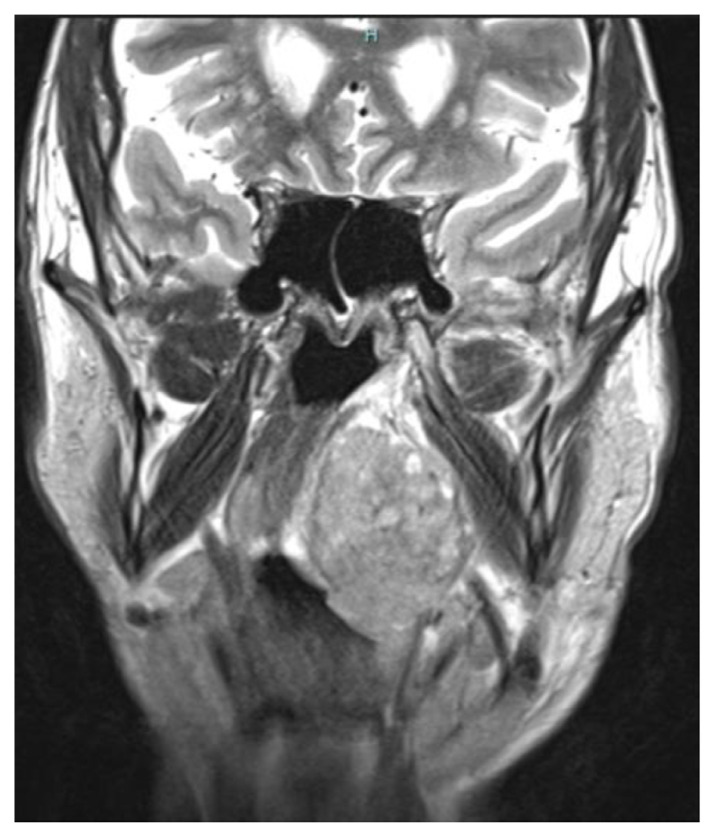
MRI coronal image before chemotherapy.

A 61-year-old male patient (Case #5), was referred to the Department of Maxillo-Facial and Oral Surgery of the San Giovanni di Dio Ruggi d’Aragona Hospital University in Salerno, Italy. On clinical examination, he presented with an extended neoformation on the left anterior tonsillar pillar. Magnetic resonance imaging (MRI) and Computerized Tomography (CT) images with and without contrast agent showed a large neoformation in the left retromolar region at the base of the tongue, also with wide extension up to the base of the cranium and neck lymph nodes causing the displacement of the structures from the midline (Fig. 1 a, b, c; and Fig. 2).

Two biopsy samples were taken as described in the methods section. The first biopsy sample was sent to the Pathology Department of the University of Salerno. The second biopsy sample was shipped to the ChemoID laboratory. Histological examination confirmed it was a poorly differentiated, basaloid, and infiltrating oral squamous cell carcinoma. In addition, an immunohistochemical exam revealed p16 positivity.

The peculiar results of the assay allowed us to give the patient the chemotherapy treatment to which he was most responsive. Table 4 shows the ChemoID test results on the CSCs enriched from the biopsy of case #5.

**Fig. 3:**
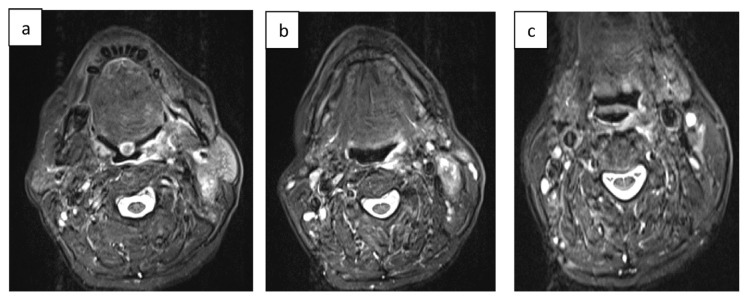
CT coronal images post-chemotherapy. a) CT image with a coronal cut on the tongue dorsum plane; b) CT image with a coronal cut on the mandibular plane; c) CT image with a coronal cut on the hyoid bone plane.

**Fig. 4:**
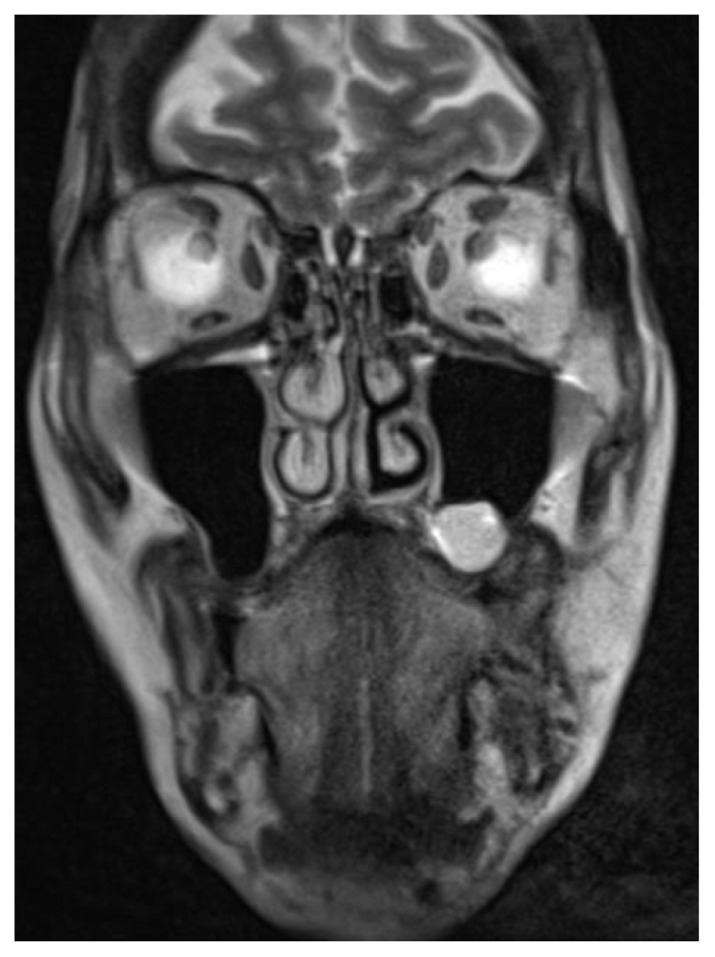
CT coronal image post-chemotherapy.

The patient was treated according to the ChemoID® assay with cisplatin 100 mg/m^2^ + 5-fluorouracil 800 mg/m^2^ + docetaxel 75 mg/m^2^ which showed a 90.5 % cell kill response. At a 2-month follow-up from the beginning of therapy, the patient had a dramatic regression of his cancer as shown in the post-chemotherapy CT images (Fig. 3 a, b, c; and Fig. 4).

## 4. Discussion

To increase the efficacy of anticancer therapy, a currently important field of research is immunotherapy, considered as the fourth pillar of the traditional surgery-chemotherapy-radiotherapy triad [12,13]. Novel approaches include the ChemoID assay, a cancer stem cell drug sensitivity assay, which pursues the goal of reducing toxicity and patients’ discomfort, improving patients’ outcomes, and limiting the development of resistant cancer cell clones [8–11].

The assay is currently used in the clinic to measure the sensitivity of cancer stem cells to different chemotherapeutics in several solid tumors including lung, breast, brain, ovarian, prostate, kidney, pancreatic cancer, and metastatic colon cancer and melanoma, however, it has only been tested recently in Head & Neck cancers [11].

Due to the variability of sensitivity to chemotherapy we observed in our patients’ cohort, its use could be extended to oral cancer to increase the effectiveness of therapy and minimize toxic effects. We have previously published another case report series of OSCC where the assay helped the oncologist choose an effective chemotherapy regimen for head and neck cancer patients and lower treatment costs by eliminating ineffective chemotherapies and unnecessary toxicity, particularly in elderly patients [11].

In the current study, we wanted to verify if the chemosensitivity of our OSCC cases was consistent with standard-of-care first-line chemotherapies indicated in the NCCN guidelines (cisplatin or 5-fluorouracil in monotherapy or in association). International guidelines recognize cisplatin 100 mg/m^2^ three times a week as the chemotherapy regimen, at different doses and times as the main reference treatment during radiotherapy performed with the most common standard fractionation [14–16].

Minimal data is available in the literature for alternatives to cisplatin and final decisions are usually based on clinicians’ experience and empirical choice. In the case of absolute exclusion of cisplatin, several alternative regimens incorporating other chemotherapies such as carboplatin, 5-fluorouracil, cetuximab, and docetaxel are available.

Results from our study confirmed the effectiveness of chemotherapies recommended in current NCCN guidelines but also showed that more precise and personalized therapy could be possible by screening chemotherapies prior to treating patients to discover more optimal treatments for each individual patient.

Our study from the analysis of the chemosensitivity reports showed that, even if the various patients were affected by the same histology of OSCC, their tumors were sensitive also to other chemotherapies than the first line of the National Comprehensive Cancer Network (NCCN) guideline-recommended cisplatin and 5-fluorouracil. It is, therefore, possible to hypothesize that, by screening each patient with the assay with those chemotherapies indicated as first-line, second-, and third-line treatments, it will be possible to improve their clinical outcome as it was demonstrated in case #5 of our cohort where an impressive reduction of the tumor was observed following ChemoID-guided-chemotherapy.

The comparison of the assay prediction for patients #1–11 in Tables 2 and 3 shows that cisplatin 100 mg/m^2^ was the most effective chemotherapy for cases # 9 and 10, whereas the other patients were found responsive to other single or combination therapies.

The combination of cisplatin 100 mg/m^2^ with 5-fluorouracil 800 mg/m^2^ and docetaxel 75 mg/m^2^ was predicted to be most effective in 6/11 (54 %) of the patients. In particular, case #5 showed a response to cisplatin 100 mg/m^2^ of only 42.8 % compared to 90.5 % of the combination of cisplatin 100 mg/m^2^+5-fluorouracil 800 mg/m^2^+docetaxel 75 mg/m^2^. By performing the ChemoID assay we were able to administer a chemotherapy cocktail to patient #5 to target more than twice as many CSCs in this patient’s tumor.

The 2020 Spanish Society of Medical Oncology (SEOM) guidelines recommend cetuximab as an alternative treatment (400 mg/m^2^ initial dose on day 8 followed by 250 mg/m^2^ weekly concomitantly) for patients with certain contraindications to cisplatin such as nephropathy, neuropathy, heart disease, and hearing loss [17]. Furthermore, the induction regimen recommended by SEOM is the TPF scheme (cisplatin 75 mg/m^2^+docetaxel 75 mg/m^2^+5-fluorouracil 750 mg/m^2^/d continuous infusion 96 h).

Taxanes (e.g. taxol, docetaxel, and paclitaxel) can also be added in combination therapy protocols [18]. The ChemoID® assay allowed us to choose the most effective type of taxane for our patient despite the negligible additional cost of the drug. For these reasons, developing and optimizing cost-effective cytotoxic chemotherapies remain important.

Patients excluded from platinum-based chemotherapy often receive external beam radiation therapy for cancer (EBRT) with cetuximab according to the National Institute for Health and Care Excellence [19]. However, EBRT is not usually the primary curative treatment of choice for tumors of the oral cavity due to local side effects. Of particular importance is the mucositis that may occur during and after treatment, causing discomfort to the patient during normal oral functions and eating [20]. Long-term pain is also possible when using high doses of radiation, which is often necessary to treat primary tumors. Osteoradionecrosis of the jaw is also a risk when irradiating the oral cavity. Neoadjuvant chemotherapy with taxanes, cisplatin, and 5-fluorouracil (TPF) is an effective combination in advanced disease for eligible patients [21].

Based on our experience, the ChemoID assay is advantageous to eliminate the choice of ineffective drugs when the patient cannot undergo a second surgery and where infiltration and extension of the neoplasm to vital structures have already occurred [11].

The main results of the present study showed that the CSC sensitivity to the various chemotherapies tested was independent of the histological type of oral cancer or tumor site. In fact, patients with OSCC responded differently to chemotherapy drugs tested by the assay.

The ChemoID assay is useful to obtain actionable information on the sensitivity of individual cancers to chemotherapy without causing additional stress to the patient because while a biopsy is sent for histological examination, another specimen can be taken for the ChemoID procedure. In addition, results can be obtained within a few days through international shipping methods able to connect countries around the world within 18–36 h.

The present communication is the second report of a successful technique we established to ensure the shipment of viable live biological samples from Europe to the United States, which allows cancer cells and CSCs to be cultured and the ChemoID test to be performed [11]. Added to this is the short time required to obtain test results, which are released within two weeks after acceptance of a viable biopsy at the ChemoID laboratory. This is a negligible amount of time, except for very advanced cancer cases that need to start treatment earlier. In this case, chemotherapy treatments could be initially started with a guideline-chosen therapy, which could be rotated to guided therapy once the results of the ChemoID assay are released.

The most important advantage of using the assay is the prospect of better outcomes as it has been shown in real-world patient data and in a randomized clinical trial of recurrent high-grade glioma patients treated with either ChemoID-guided chemotherapy or chemotherapies chosen by the physician [22–24].

Looking at the ChemoID reports from our patients, platinum-based guideline therapies at the highest dose (100 mg/m^2^) resulted in intermediate-high cell kill sensitivity; however, better efficacy could have been achieved through a personalized approach following ChemoID indications.

Another advantage of the ChemoID assay is the possibility of repeating the procedure starting from a new biopsy in case of tumor recurrence. This is because repeated ineffective treatments cause resistant cancer stem cell selection, and repeated testing could help resolve the dilemma of identifying the most effective therapies for the selected resistant CSCs. Further interventional prospective studies are needed on a larger cohort of patients to determine the clinical validity of the ChemoID assay to treat head & neck cancer.

## 5. Conclusions

One way to improve the outcome of patients affected by advanced cancer is to identify all measures and procedures aimed at creating greater efficiency, effectiveness, precision, and fast delivery of services to support the National Healthcare System in the event of stress or crisis. In this way, the provision of essential therapies could be preserved through the selection of less impacting procedures, that are still able to offer an adequate assistance response in a Public Health System delocalized to the patient’s home and in a network of outpatient clinics spread throughout the territory.

Our results highlight the potential of the ChemoID assay to individually target therapy for head & neck cancer patients, increasing the effectiveness of chemotherapy interventions.

Viable biopsy samples were transported from Europe to the ChemoID laboratory in the United States within 18–36 h, highlighting this novel test’s healthcare potential. Treatments with expensive targeted anti-cancer drugs are not always feasible due to socio-economic and health disparity issues in the United States and around the world. The ability of the assay to personalize chemotherapy selection is a promising way to provide more affordable treatment for head & neck cancer patients who may need chemotherapy treatments.

Larger studies are needed to validate these observations. The ChemoID assay is versatile, allowing it to be expanded to include other new agents. Further studies will allow the testing of new chemotherapy drugs for the clinical management of head & neck cancer with the assay.

## Figures and Tables

**Table 1:** Patient series demographics.

Case	Age	Sex	Site	Origin	Histology	Smoking	Alcohol
1	49	M	Tongue	Mucosa	OSCC	Yes	Yes
2	82	M	Oral mucosa	Mucosa	OSCC	Yes	Yes
3	78	M	Lower lip	Mucosa	OSCC	Yes	Yes
4	79	M	Oral mucosa	Mucosa	OSCC	Yes	Yes
5	61	M	Left anterior tonsillar pillar	Mucosa	OSCC	Yes	Yes
6	50	M	Mouth Floor	Mucosa	OSCC	Yes	Yes
7	75	M	Retromolar space	Mucosa	OSCC	Yes	No
8	79	M	Retromolar space	Mucosa	OSCC	Yes	No
9	84	F	Tongue right margin	Mucosa	OSCC	No	No
10	81	M	Cheek	Mucosa	OSCC	Yes	No
11	68	M	Tuber Maxillae	Mucosa	OSCC	Yes	No

**Table 2:** Percentage cell-kill response of CSCs enriched from biopsies of patients affected by oral squamous cell carcinoma to single or combination chemotherapy agents. The top indicates the patient number. On the left are the chemotherapies and doses tested.

	1	2	3	4	5	6	7	8	9	10	11
Paclitaxel 40 mg/m^2^	<10%										
Paclitaxel 175 mg/m^2^	<10%										
Carboplatin 70 mg/m^2^	<10%		<10%	<10%	<10%	<10%	<10%		<10%		
Carboplatin 100 mg/m^2^	<10%	25.5%	<10%	<10%	<10%	<10%	<10%	18.6%	<10%	49.6%	10.8%
Cisplatin 50 mg/m^2^	27.0%		52.6%	37.9%	35.3%	67.2%	46.3%		53.4%		
Cisplatin 100 mg/m^2^	34.2%	73.6%	88.1%	85.3%	42.8%	86.4%	76.8%	78.3%	78.6%	61.3%	42.9%
Docetaxel 75 mg/m^2^	22.2%	87.4%	29.3%	29.2%	49.2%	54.1%	25.0%	<10%	25.6%	<10%	<10%
Paclitaxel 40 mg/m^2^	<10%		32.9%	10.5%	26.2%	<10%	27.9%		<10%	<10%	
Paclitaxel 175 mg/m^2^	<10%	17.1%	26.8%	<10%	46.6%	16.5%	21.0%	55.0%	<10%	41.4%	<10%
5-Fluorouracil 600 mg/m^2^	<10%		<10%	<10%	<10%	<10%	<10%		<10%	<10%	
5-Fluorouracil 800 mg/m^2^				14.6%	<10%	11.2%	<10%		<10%		
5-Fluorouracil 1000 mg/m^2^	<10%	30.0%	<10%	12.5%				28.1%		16.6%	<10%
Bleomycin 15 mg/m^2^				<10%				<10%		38.5%	<10%
Methotrexate 40 mg/m^2^											<10%
Cetuximab 400 mg/m^2^											<10%
Vinorelbine 25 mg/m^2^								26.8%		<10%	
Erlotinib 150 mg								<10			
Ifosfamide 5000 mg/m^2^		65.2%									
Gemcitabine 1000 mg/m^2^		34.5%						31.0%		%	
Gemcitabine 1000 mg/m^2^ + Vinorelbine 25 mg/m2								18.5%		<10%	
Paclitaxel 40 mg/m^2^ + Cisplatin 50 mg/m^2^	30.0%		58.0%	53.8%	40.5%	78.3%	43.1%		57.7%	16.5%	
Paclitaxel 175 mg/m^2^ + Cisplatin 100 mg/m^2^		77.7%						90.5%			48.6%
Paclitaxel 40 mg/m^2^ + Carboplatin 100 mg/m^2^	<10%		<10%	22.8%	<10%	17.9%	<10%		<10%	24.2%	
5-Fluorouracil 600 mg/m^2^ + Carboplatin 70 mg/m^2^	<10%		<10%	21.4%	<10%	15.9%	<10%	52.5%	<10%	31.8%	
5-Fluorouracil 800 mg/m^2^ + Cisplatin 50 mg/m^2^	27.0%		58.0%	42.7%	52.4%	72.3%	63.6%		45.2%		
5-Fluorouracil 1000 mg/m^2^ + Cisplatin 100 mg/m^2^		70.1%						78.0%		<10%	38.9%
Cisplatin 100 mg/m^2^ + Docetaxel 75 mg/m^2^		93.5%						72.8%			42.5%
Carboplatin 100 mg/m^2^ + Docetaxel 75 mg/m^2^								26.8%			
Cisplatin 100 mg/m^2^ + 5-Fluorouracil 800 mg\m^2^ + Docetaxel 75 mg\m^2^	94.0%		96.2%	100.0%	90.5%	94.3%	90.6%		58.7%	39.5%	
Cisplatin 100 mg/m^2^ + 5-Fluorouracil 1000 mg\m^2^ + Docetaxel 75 mg\m^2^		89.3%									
Cisplatin 100 mg/m^2^ + 5-Fluorouracil 600 mg\m^2^ + Paclitaxel 175 mg\m^2^	47.3%		62.4%	65.9%	61.5%	91.8%	58.5%		71.6%		
Cisplatin 100 mg/m^2^ + 5-Fluorouracil 1000 mg\m^2^ + Paclitaxel 175 mg\m^2^		74.4%								10.4%	48.7%

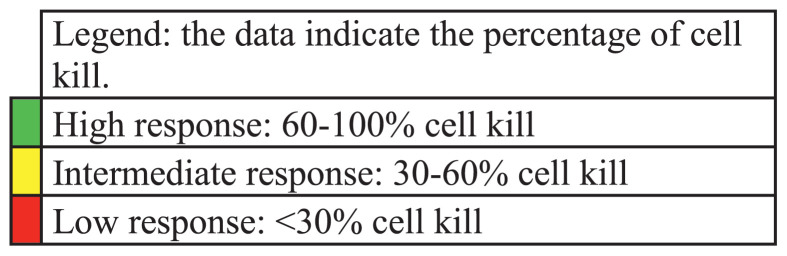

**Table 3:** The highest cell kill detected by the ChemoID® assays on CSCs in each patient sample.

N. Case	Drug(s)	Percentage cell kill
1	cisplatin 100mg/m2 + 5-fluorouracil 800mg/m2 + docetaxel 75mg/m2	94.0
2	cisplatin 100mg/m2 + docetaxel 75mg/m2	93.5
3	cisplatin 100mg/m2 + 5-fluorouracil 800mg/m2 + docetaxel 75mg/m2	96.2
4	cisplatin 100mg/m2 + 5-fluorouracil 800mg/m2 + docetaxel 75mg/m2	100.0
5	cisplatin 100mg/m2 + 5-fluorouracil 800mg/m2 + docetaxel 75mg/m2	90.5
6	cisplatin 100mg/m2 + 5-fluorouracil 800mg/m2 + docetaxel 75mg/m2	94.3
7	cisplatin 100mg/m2 + 5-fluorouracil 800mg/m2 + docetaxel 75mg/m2	90.6
8	paclitaxel 175mg/m2 + cisplatin100mg/m2	90.5
9	cisplatin 100mg/m2	78.6
10	cisplatin 100mg/m2	61.3
11	cisplatin 100mg/m2 + 5-fluorouracil 1000mg/m2 + paclitaxel 175mg/m2	48.7

**Table 4:** Percentage cell-kill response of CSCs enriched from the OSCC biopsy of patient #5 to single or combination chemotherapy agents.

Cisplatin 100 mg/m^2^ + 5-Fluorouracil 800 mg/m^2^ + Docetaxel 75 mg/m^2^	90.5%
Cisplatin 100 mg/m^2^ + 5-Fluorouracil 600 mg/m^2^ + Paclitaxel 175 mg/m^2^	61.5%
5-Fluorouracil 800 mg/m^2^ + Cisplatin 50 mg/m^2^	52.4%
Docetaxel 75 mg/m^2^	49.2%
Paclitaxel 175 mg/m^2^	46.6%
Cisplatin 100 mg/m^2^	42.8%
Paclitaxel 40 mg/m^2^ + Cisplatin 50 mg/m^2^	40.5%
Cisplatin 50 mg/m^2^	35.3%
Paclitaxel 40 mg/m^2^	26.2%
Carboplatin 70 mg/m^2^	<10%
Carboplatin 100 mg/m^2^	<10%
5-Fluorouracil 600 mg/m^2^	<10%
5-Fluorouracil 800 mg/m^2^	<10%
Paclitaxel 40 mg/m^2^ + Carboplatin 100 mg/m^2^	<10%
5-Fluorouracil 600 mg/m^2^ + Carboplatin 70 mg/m^2^	<10%

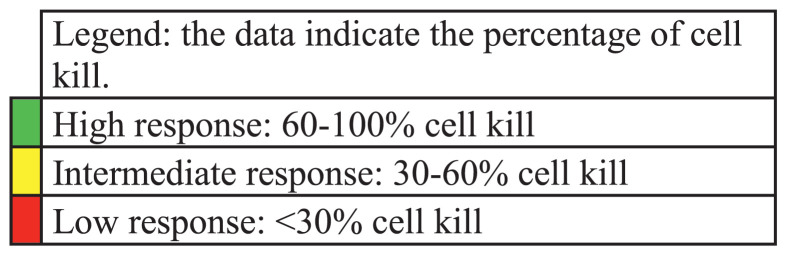

## Data Availability

Data is available upon reasonable request.

## References

[1] Ferreira E Costa R Leao MLB Sant’Ana MSP Mesquita RA Gomez RS Oral squamous cell carcinoma frequency in young patients from referral centers around the world Head Neck Pathol 2022 16 755 62 35316511 10.1007/s12105-022-01441-w PMC9424469

[2] Goyal N Hennessy M Lehman E Lin W Agudo A Ahrens W Risk factors for head and neck cancer in more and less developed countries: analysis from the INHANCE consortium Oral Dis 2023 May 29 4 1565 78 35322907 10.1111/odi.14196

[3] George R Neralla M Rajan J Haque AE Kumar SP Metastatic tumour to the mandible - a diagnostic and management dilemma Cureus 2019 11 e5093 31516800 10.7759/cureus.5093 PMC6721909

[4] Adorno-Cruz V Kibria G Liu X Doherty M Junk DJ Guan D Cancer stem cells: targeting the roots of cancer, seeds of metastasis, and sources of therapy resistance Cancer Res 2015 75 924 9 25604264 10.1158/0008-5472.CAN-14-3225 PMC4359955

[5] Shahoumi LA Oral cancer stem cells: therapeutic implications and challenges Front Oral Health 2021 2 685236 35048028 10.3389/froh.2021.685236 PMC8757826

[6] Miyoshi N Haraguchi N Mizushima T Ishii H Yamamoto H Mori M Targeting cancer stem cells in refractory cancer Regen Ther 2021 17 13 9 33598510 10.1016/j.reth.2021.01.002 PMC7868918

[7] Shin KH Kim RH An updated review of oral cancer stem cells and their stemness regulation Crit Rev Oncog 2018 23 189 200 30311574 10.1615/CritRevOncog.2018027501 PMC6436811

[8] Howard CM Bush S Zgheib NB Lirette ST Cortese A Mollo A Cancer stem cell assay for the treatment of platinum-resistant recurrent ovarian cancer HSOA J Stem Cells Res Dev Ther 2021 7 10.24966/srdt-2060/100076 PMC8597976 34796266

[9] Ranjan T Howard CM Yu A Xu L Aziz K Jho D Cancer stem cell chemotherapeutics assay for prospective treatment of recurrent glioblastoma and progressive anaplastic glioma: a single-institution case series Transl Oncol 2020 13 100755 32197147 10.1016/j.tranon.2020.100755 PMC7078520

[10] Howard CM Valluri J Alberico A Julien T Mazagri R Marsh R Analysis of chemopredictive assay for targeting cancer stem cells in glioblastoma patients Transl Oncol 2017 10 241 54 28199863 10.1016/j.tranon.2017.01.008 PMC5310181

[11] Cortese A Pantaleo G Amato M Lawrence L Mayes V Brown L A new complementary procedure for patients affected by head and neck cancer: chemo-predictive assay Int J Surg Case Rep 2016 26 42 6 27449762 10.1016/j.ijscr.2016.07.013 PMC4963245

[12] Clarke E Eriksen JG Barrett S The effects of PD-1/PD-L1 checkpoint inhibitors on recurrent/metastatic head and neck squamous cell carcinoma: a critical review of the literature and meta-analysis Acta Oncol 2021 60 1534 42 34410881 10.1080/0284186X.2021.1964699

[13] Belgioia L Becherini C Bacigalupo A Bonomo P Chemoimmunotherapy and radiation in locally advanced head and neck cancer: where do we stand? Oral Oncol 2022 127 105773 35217401 10.1016/j.oraloncology.2022.105773

[14] Hennequin C Guillerm S Quero L Combination of chemotherapy and radiotherapy: a thirty years evolution Cancer Radiother 2019 23 662 5 31473087 10.1016/j.canrad.2019.07.157

[15] Szturz P Cristina V Herrera Gomez RG Bourhis J Simon C Vermorken JB Cisplatin eligibility issues and alternative regimens in locoregionally advanced head and neck cancer: recommendations for clinical practice Front Oncol 2019 9 464 31245288 10.3389/fonc.2019.00464 PMC6579895

[16] Carlsson L Bratman SV Siu LL Spreafico A The cisplatin total dose and concomitant radiation in locoregionally advanced head and neck cancer: any recent evidence for dose efficacy? Curr Treat Options Oncol 2017 18 39 28555374 10.1007/s11864-017-0482-0

[17] Mesia R Iglesias L Lambea J Martinez-Trufero J Soria A Taberna M SEOM clinical guidelines for the treatment of head and neck cancer (2020) Clin Transl Oncol 2021 23 913 21 33635468 10.1007/s12094-020-02533-1 PMC8057973

[18] Joseph B Vishwanath L Venugopal BK Radiosensitization in head and neck cancer: do we have an alternative to platins? Role of taxanes Oral Surg Oral Med Oral Pathol Oral Radiol 2014 117 324 8 24388535 10.1016/j.oooo.2013.10.006

[19] Al-Saleh K El-Sherify M Safwat R Elbasmy A Shete J Hussein A Phase II/III randomized controlled trial of concomitant hyperfractionated radiotherapy plus cetuximab (Anti-EGFR antibody) or chemotherapy in locally advanced head and neck cancer Gulf J Oncolog 2019 1 6 12 31242976

[20] Vedasoundaram P Raghava Ks A Periasamy K Selvarajan GKS Kandasamy SRS Kumar A The effect of high dose rate interstitial implant on early and locally advanced oral cavity cancers: update and long-term follow-up study Cureus 2020 12 e7910 32494525 10.7759/cureus.7910 PMC7263712

[21] Ghi MG Paccagnella A D’Amanzo P Mione CA Fasan S Paro S Neoadjuvant docetaxel, cisplatin, 5-fluorouracil before concurrent chemoradiotherapy in locally advanced squamous cell carcinoma of the head and neck versus concomitant chemoradiotherapy: a phase II feasibility study Int J Radiat Oncol Biol Phys 2004 59 481 7 15145166 10.1016/j.ijrobp.2003.10.055

[22] Ranjan T Sengupta S Glantz M Green R Yu A Aregawi D A multi-institutional randomized clinical trial comparing assay - guided chemotherapy with physicianchoice treatment for recurrent high-grade glioma (Nct03632135) Neuro Oncol 2022 24 73 4

[23] Ranjan T Sengupta S Glantz M Green R Yu A Aregawi D Multi-institutional randomized phase 3 trial comparing cancer stem cell-targeted versus physician-choice treatments in patients with recurrent high-grade gliomas (NCT03632135) J Clin Oncol 2022 40 34388022

[24] Ranjan T Sengupta S Glantz M Green R Yu A Aregawi D Cancer stem cell assay-guided chemotherapy improves survival of patients with recurrent glioblastoma in a randomized trial Cell Rep Med 2023 4 101025 37137304 10.1016/j.xcrm.2023.101025 PMC10213810

